# Interplay of Systemic Immune-Inflammation Index and Serum Klotho Levels: Unveiling a New Dimension in Rheumatoid Arthritis Pathology

**DOI:** 10.7150/ijms.89569

**Published:** 2024-01-01

**Authors:** Jinlong Zhao, Yifan Jia, Lingfeng Zeng, Hetao Huang, Guihong Liang, Kunhao Hong, Bangxin Sha, Minghui Luo, Jun Liu, Weiyi Yang

**Affiliations:** 1 The Second Clinical College/State Key Laboratory of Traditional Chinese Medicine Syndrome of Guangzhou University of Chinese Medicine, Guangzhou 510405, China.; 2 The Second Affiliated Hospital of Guangzhou University of Chinese Medicine (Guangdong Provincial Hospital of Chinese Medicine), Guangzhou 510120, China.; 3 The Fifth Clinical College of Guangzhou University of Chinese Medicine, Guangzhou 510405, China.; 4 The Research Team on Bone and Joint Degeneration and Injury of Guangdong Provincial Academy of Chinese Medical Sciences, Guangzhou 510120, China.; 5 Guangdong Second Chinese Medicine Hospital (Guangdong Province Engineering Technology Research Institute of Traditional Chinese Medicine), Guangzhou 510095, China.

**Keywords:** association, systemic immune-inflammation index, SII, rheumatoid arthritis, Klotho

## Abstract

**Aim:** The association between the systemic immune-inflammation index (SII) and serum Klotho concentrations (pg/ml) in patients with rheumatoid arthritis (RA) has not been elucidated. The purpose of this study was to clarify the relationship between the SII and serum Klotho concentrations in RA patients.

**Methods**: All data come from the National Health and Nutrition Examination Survey (NHANES) database in the United States, which included 982 RA patients (age range: 40 to 79 years). The measurement data of the SII and serum Klotho are all from the NHANES mobile examination centre. We constructed a multivariate linear regression model to evaluate the association between the SII and serum Klotho levels in RA patients and conducted a subgroup analysis to test the stability of the statistical results. **Results:** Multivariate linear regression results indicated a negative linear relationship between the SII and serum Klotho concentrations in RA patients (β = -6.33, 95% CI [confidence interval]: -10.15 to -2.53). Compared to the quartile 1 group, the quartile 4 group was associated with significantly lower (*P*<0.001) serum Klotho concentrations (β = -120.93, 95% CI: -174.84 to -67.02). Compared with the quartile 1 group, with the increase in the SII, the β value showed a decreasing trend (*P*_trend_ < 0.001). The subgroup analysis showed that none of the covariates affected the stability of these results (all* P* for interaction ≥ 0.05).

**Conclusion:** There is a significant negative linear association between the SII and serum Klotho concentrations in RA patients. The SII can serve as a predictive indicator of serum Klotho concentrations in RA patients, and Klotho may be a potential anti-inflammatory target for RA treatment.

## Introduction

Rheumatoid arthritis (RA) is a common systemic autoimmune disease that is characterized by chronic systemic inflammation 1,2. The global prevalence of RA is approximately 1% 3. The clinical manifestations of RA are mostly symmetrical small joint inflammation, dysfunction, stiffness, and deformity, which are believed to be related to inflammatory factor infiltration, cell proliferation, soft tissue swelling, bone erosion, and immune overactivation, and lesions can also affect extra-articular tissues such as the heart, lungs, pleura, and eyes 4-6. The pathogenesis of RA has not been fully elucidated, but it is believed to be closely related to genetic, environmental, and epigenetic modifications 6,7. The pathogenesis of RA involves the activation of inflammatory cytokines such as tumour necrosis factor-α (TNF-α), interleukin-6 (IL-6), and interleukin-7 (IL-7), leading to bone and cartilage destruction 8-9. At present, there are no curative drugs or measures for RA. Nonsteroidal anti-inflammatory drugs (NSAIDs), disease-relieving anti-rheumatic drugs (DMARDs), glucocorticoids (GCs), and biological agents are commonly used in clinical practice to alleviate RA progression 10,11. Based on the current research on RA, we believe that the level of inflammation is closely related to the clinical manifestations, diagnosis, and treatment of RA. The systemic immune inflammation index (SII) is calculated based on the counts of neutrophils, platelets, and lymphocytes in peripheral blood 12. The SII is believed to reflect the degree of systemic inflammation and the immune status of the body. In addition, the SII can be easily calculated and obtained through routine blood testing data, which has the advantages of simplicity, efficiency, and low cost.

Klotho, a transmembrane protein that is highly expressed in renal tubular epithelial cells, is considered a longevity gene and is associated with antioxidant, anti-inflammatory, anti-apoptosis, and anti-ageing effects 13,14. Research has shown that high expression of the Klotho gene can prolong the lifespan of mice and enhance their antioxidant stress ability, indicating that Klotho is a gene with anti-apoptotic and anti-ageing effects 15,16. Research has shown that the Klotho expression level of CD4+ T lymphocytes is significantly reduced in RA patients 17,18. Li et al. found that a decrease in Klotho expression in macrophages and peripheral blood monocytes can lead to an increase in the expression of the inflammatory mediators MMP-9, IL-6, and TNF-α 19. Therefore, we believe that the immune level and inflammatory status of RA patients may affect their serum Klotho concentrations, but the relationship between those variables has not yet been elucidated. We hypothesize that there is a dose‒response relationship between the SII and serum Klotho concentrations in RA patients. To confirm our hypothesis, we will apply data from the National Health and Nutrition Examination Survey (NHANES) database in the United States to explore the relationship between the SII and serum Klotho levels in RA patients. Our expectation is that this study can elucidate the relationship between immune inflammation levels and serum Klotho concentrations in RA patients and provide a new perspective for further understanding the role of serum Klotho levels in the pathological progression of RA.

## Methods

### Data sources and participants

The raw data used in this study were obtained from the NHANES database. The NHANES was a cross-sectional survey conducted by the Centers for Disease Control and Prevention (CDC) in the United States 20. NHANES data were collected using a stratified multistage sampling design from a nationally representative sample of American civilians, with the aim of monitoring the health and nutritional status of adults and children across the United States 20. Due to the independent variables and endpoint outcomes of this study being the SII and serum Klotho concentrations, we only selected the five survey periods from 2007 to 2016 that had sufficient measurement data to calculate these two variables. All participants signed an informed consent form 21; NHANES has been approved by the Ethics Review Committee of the National Center for Health Statistics in the United States 21.

**Figure 1:**
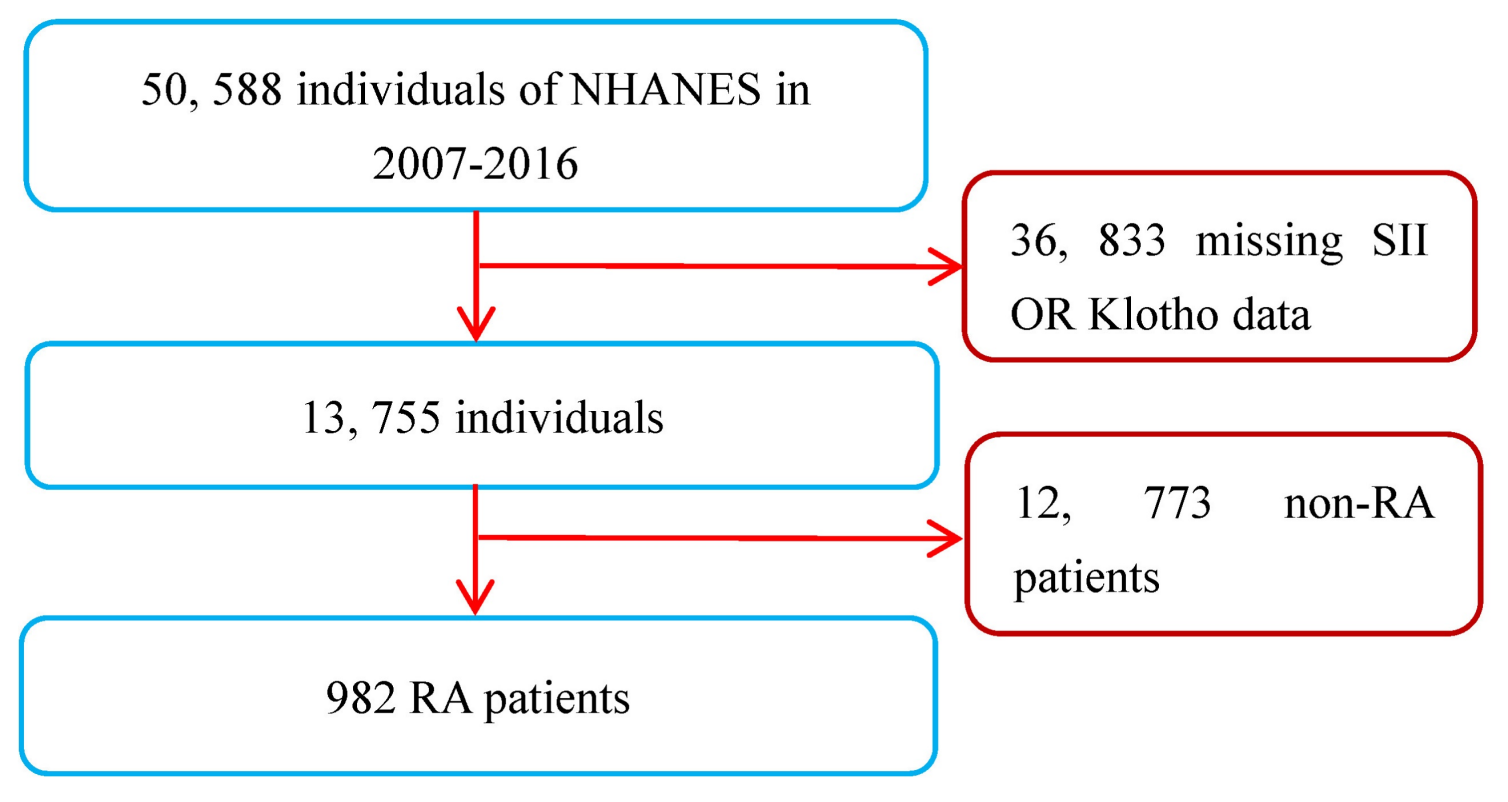
Flow chart of study patients. Abbreviations: NHANES: National Health and Nutrition Examination Survey; RA: rheumatoid arthritis; SII: systemic immune-inflammation index.

A total of 50,588 participants were sampled and participated in the survey from 2007 to 2016. A total of 982 RA patients were found in personal interview data on various health status self-reports, and their diagnosis was mainly based on their previous diagnosis of RA by doctors 20. As the measurement data of the SII and serum Klotho concentrations were key variables in this study, all RA patients lacking these two variables were not included for further analysis. However, participants who were only missing covariate data were not excluded, and those missing values were set as dummy variables in the dataset. Apart from this, there were no other exclusion or inclusion criteria. Ultimately, a total of 982 RA patients (age range: 40 to 79 years) were included in this study. The inclusion and exclusion process of the research subjects is shown in Figure 1.

### Measurement of the SII

The SII is an immune inflammation indicator calculated based on the counts of neutrophils, platelets, and lymphocytes in peripheral blood. The calculation formula is SII = platelet count × neutrophil count/lymphocyte count 12. Blood samples from all RA patients were collected at the NHANES Mobile Examination Center (MEC). The instruments used for whole blood cell count analysis were a Coulter HMX (Coulter Electronics Ltd., Bedfordshire, UK) 22 or Beckman Coulter DXH 800 (Beckman Coulter, Brea, CA, USA) 23. The laboratory method for whole blood cell counting can be found on the NHANES website.

### Serum soluble Klotho concentrations

We collected serum soluble Klotho (pg/ml) data that was obtained from RA patients as part of the NHANES from 2007 to 2016. Whole blood samples were obtained from participants, and serum was prepared by MEC centrifugation. The serum samples were stored at -80 °C by the CDC in Atlanta, Georgia (GA). The measurement of serum Klotho concentrations was carried out using a commercially available ELISA kit (IBL International, Japan) from a CDC certified laboratory 24. The Klotho concentrations of all serum samples were analysed twice, and the average of these two measured concentrations was used as the final measurement value. In addition, the manufacturer declares that the sensitivity of the Klotho concentration measurement is 6.15 pg/mL. In fact, the concentration measurement sensitivity of the laboratory was 4.33 pg/mL. The sample information, experimental measurement details, and quality control of the Klotho concentration measurements can be found on the NHANES official website 25.

### Assessment of covariates

The selection of covariates in this study was mainly based on the literature and the variables recognized by the academic community 26-27. The selected covariates primarily included sociodemographic factors: age (years), sex, race (non-Hispanic white, non-Hispanic black, American-Mexican, and others), and body mass index (BMI). We divided the patients into three groups based on BMI values: <25.0, 25.0-29.9, and ≥ 30 kg/m^2^. In addition, we extracted six diseases, including diabetes 28, chronic kidney disease (CKD) 29, hypertension 30, hyperlipidaemia 31, chronic obstructive pulmonary disease (COPD) 32, and coronary heart disease (CHD) 33, as covariates. The diagnosis of these six diseases was based on clear diagnostic criteria 28-33 and was matched with measurement indicators in the NHANES database, or each RA patient included in this study voluntarily reported taking disease-related drugs, such as hypoglycaemic drugs and antihypertensive drugs, or participants reported having been diagnosed by a doctor.

### Statistical analyses

The data analysis of this study was carried out according to the statistical guidelines of the CDC 34. All data extraction and analysis were conducted using R software (version 4.2.2, http://www.R-project.org, The R Foundation) and EmpowerStats software (version 4.1, www.empowerstats.com, X&Y solutions, Inc. Boston, MA, USA). All tests were bilateral and statistical significance was determined a priori (α= 0.05). Continuous variables are represented by weighted mean and standard deviation (SD); categorical variables are expressed by frequency and weighted percentage. We used a weighted linear regression model to evaluate the relationship between the SII and serum Klotho concentrations in RA patients. Considering that the absolute value of the SII is relatively large and to enhance the statistical efficiency of the data and facilitate clinical application, we divided all SII values by 100 to use as the raw data. Therefore, in this study, an increase of one unit in the SII means an increase of 100 in the absolute value of the SII. To test the stability of the influence of the SII on Klotho levels in different regression models, we divided the SII value into four groups according to the quartile. The SII was entered into the model as a continuous variable and as a categorical variable (quartile grouping). To further test the stability of the relationship between the SII and Klotho, we constructed three regression models to gradually adjust for confounding factors: Model 1 did not adjust for covariates; Model 2 adjusted for age, sex, and race; and Model 3 further adjusted for BMI, diabetes, CKD, tension, COPD, CHD, and hyperlipidaemia on the basis of Model 2. We further conducted interaction tests (i.e., subgroup analysis) on all the covariates, which will be beneficial for the comprehensive evaluation of the reliability of our research conclusions.

## Results

### Baseline characteristics of study individuals

A total of 982 RA patients were included in this study, with an age range of 40-79 years old. The weighted average serum Klotho concentrations in the SII Q1-Q4 groups were 909.36, 828.83, 818.91, and 781.16, respectively, and the differences between the groups were statistically significant (*P*<0.001). In general, the SII quartiles had significant differences in race/ethics, CKD, hypertension, neutrophil count, lymphocyte count, and platelet count. The basic characteristics of the study population grouped according to SII quartiles are shown in Table 1. Compared with the non-RA group, the SII value of RA patients was higher, while the Klotho level was lower, and the differences were statistically significant (*P*<0.001 and *P*<0.05, respectively). The characteristics of the RA and non-RA populations can be found in Supplementary Table 1.

### Association between the SII and Klotho

We constructed three linear regression models to explore the independent effect of SII on serum Klotho (Table 2). All covariate-adjusted models (Model 3) showed a significant correlation between a higher SII and lower serum Klotho concentrations (β = -6.33, 95% CI [confidence interval]: -10.15 to -2.53). Model 3 results showed that compared to the Q1 group, the serum Klotho concentrations in the Q4 group was lower (β = -120.93, 95% CI: -174.84 to -67.02), with statistically significant differences (P<0.001). Compared with the Q1 group, with the increase in SII levels, the β values of the Q2 to Q4 groups showed a downwards trend (*P*_trend_ < 0.001). The results of the single-factor and multivariate linear regression analyses (Table 2) indicated that higher SII values are correlated with lower serum Klotho concentrations, indicating a negative linear relationship between the two.

### Subgroup analysis

To further test the stability of this regression model, we conducted interaction tests on all covariates (Table 3). We found a significant inverse association between higher SII values and lower Klotho concentrations in serum, and the association was not affected by age, sex, race/ethnicity, BMI, diabetes, CKD, hypertension, COPD, CHD, or hyperlipidaemia (all *P* for interaction ≥ 0.05).

## Discussion

The role of the Klotho gene in RA has rarely been studied, but we believe that Klotho protein may have significant potential application value in alleviating the pathological progression of RA. Klotho is an antiaging gene widely expressed in the kidneys, brain tissue, and parathyroid glands 35. Its encoded secretory protein can enter the bloodstream in the form of hormonal substances and exert antioxidant, anti-inflammatory, and anti-apoptotic functions. Cytokines are important regulators of physiological and pathological processes in the body, reflecting the disease status of RA and mediating bone destruction in RA patients 36-38. Various inflammatory cytokines can be involved, such as interleukin 17 (IL-17), in the synovial inflammatory response of RA patients 39. The SII has been widely used to evaluate immune system functional status or inflammation levels and has been proven to have good clinical application value in diagnosing diseases or serum factor levels 40-42. However, the relationship between the SII and serum Klotho concentrations in RA patients has not been studied, which will hinder our further understanding of the potential association between inflammation levels and Klotho. To the best of our knowledge, our research provides the first valuable data on this topic. In this cross-sectional study, we found a negative linear relationship between the SII and serum Klotho concentration in RA patients, indicating that higher levels of immune inflammation in RA patients are associated with lower Klotho levels. Based on the results of this study, we propose the hypothesis that if there is a causal relationship between the SII and Klotho concentrations, then promoting the secretion of endogenous Klotho protein or exogenous supplementation with Klotho protein may provide significant clinical value in the anti-inflammatory treatment of RA.

To further test the stability of the statistical results, we conducted a stratified analysis on all covariates. Notably, the inverse linear association between the SII and serum Klotho concentrations in RA patients was not affected by all covariates, thus indicating the relative stability of the conclusion in this study.

RA mainly involves inflammatory reactions within the synovium of joints, and its basic pathological changes include synovitis of multiple joints. Long-term inflammatory reactions lead to the destruction of joint cartilage and bone structures 43, 44, ultimately leading to joint deformities and loss of function. The inflammatory response is the core link in the pathogenesis of RA, and macrophages in synovitis tissue play an important role in the inflammatory response 45, 46. Research shows that the inflammatory environment of RA patients secretes a large number of inflammatory cytokines by macrophages and recruits immune cells to migrate to joints, thereby promoting the occurrence of joint damage 47, 48. We believe that the SII can comprehensively reflect the level of immune inflammation in RA patients, but the relationship between the SII and the progression of RA still needs further research. Klotho has been reported to protect tissues from damage through antioxidant and anti-inflammatory effects 27, and aerobic exercise has been shown to increase serum Klotho levels and delay RA progression. Russell et al. concluded that although the exact mechanism of Klotho in CD4+ cell function is not yet clear 49, it may be involved in the anti-inflammatory process of young healthy individuals, while its concentration may decrease in healthy elderly individuals and RA patients. An animal experiment 50 showed that exogenous Klotho supplementation could reduce the levels of reactive oxygen species (ROS) in injured kidney tissues and superoxide dismutase (SOD) and malondialdehyde (MDA) in serum as well as the production of IL-6 and TNF-α. In addition, another study showed that Klotho can inhibit the activation of NF-κB-related inflammatory pathways 50. Han et al. 51 reported that higher levels of Klotho in serum are associated with lower levels of neutrophils, which may be related to the anti-inflammatory activity of Klotho proteins. Kaszubowska et al. 52 found a positive correlation between serum Klotho levels and lymphocyte secretion, which may be related to Klotho's ability to maintain immune homeostasis. In addition, platelet volume is believed to be positively correlated with serum Klotho concentrations 53, and Klotho has been proven to play a protective role in the inflammatory process. Wu et al.'s study confirmed that lower levels of Klotho are associated with inflammation 53, especially in clinical settings where white blood cell counts increase and platelet volume decreases. It is worth noting that the physiological mechanism of Klotho in regulating neutrophils, lymphocytes, and platelets is still lacking and will depend on further exploration in future research. Based on the conclusions of this study, we believe that Klotho may be a key anti-inflammatory target in the treatment of RA. Although there is still a lack of research or drug development on Klotho protein as an anti-inflammatory target for RA, we believe that Klotho protein will need to be considered in the anti-inflammatory treatment of RA.

### Limitations

It is undeniable that there are several deficiencies in this study, and these deficiencies should be appropriately considered when applying this conclusion. The design of this study is a cross-sectional study, which means that the causal relationship has not been fully confirmed here. Therefore, the determination of causality still needs to be confirmed by further prospective cohort studies or Mendelian randomized studies in the future, which will be the key to the application of Klotho in the research of RA targets. Second, due to the lack of measurement data for SII or serum Klotho protein in some patients, we had to exclude these RA patients from participation in this study. Due to the absence of these samples, there may be potential bias in the conclusions of this study. In addition, this study is based on the NHANES database, and not all indicators related to RA can be obtained, such as rheumatoid factor (RF), anti-citrullinated protein antibodies (ACPA), DAS28 score, and erythrocyte sedimentation rate (ESR). Due to the lack of these data, we are unable to have a clearer understanding of these indicators and their characteristics in RA patients. Finally, although we adjusted for multiple confounding factors that may affect the conclusions of this study, there may still be other key confounding factors that have not been adjusted due to the level of understanding of the disease and limitations of the study design.

## Conclusions

In this study, we found a significant negative linear relationship between the SII and serum Klotho concentration in RA patients, indicating a correlation between higher levels of immune inflammation and lower Klotho concentration. The SII can serve as a predictive indicator of serum Klotho concentration in RA patients, and Klotho may serve as a potential anti-inflammatory target for RA treatment. The causal relationship between the SII and serum Klotho concentration still needs further prospective cohort studies or Mendelian randomized studies for verification.

## Supplementary Material

Supplementary table.

## Figures and Tables

**Table 1:** Characteristics of individuals by SII quartile from NHANES (2007-2016).

	SII levels (N = 982)				
Characteristic	Quartile 1	Quartile 2	Quartile 3	Quartile 4	*P-*Value
Age, years					0.588
40-59	111 (56.45)	100 (54.54)	103 (51.80)	109 (54.41)	
60-69	85 (27.46)	90 (25.33)	94 (31.64)	76 (25.50)	
70-79	50 (16.09)	55 (20.13)	48 (16.55)	61 (20.09)	
Sex					0.983
Male	104 (40.92)	89 (39.40)	101 (39.94)	106 (39.33)	
Female	142 (59.08)	156 (60.60)	144 (60.06)	140 (60.67)	
Race/ethnicity					0.021
Non-Hispanic White	102 (24.89)	73 (15.74)	65 (15.60)	53 (10.61)	
Non-Hispanic Black	75 (60.43)	88 (67.03)	90 (66.43)	113 (69.27)	
American Mexican	34 (6.36)	44 (8.02)	46 (8.70)	38 (9.08)	
Others	35 (8.32)	40 (9.21)	44 (9.27)	42 (11.04)	
BMI					0.59
<25	45 (21.27)	44 (18.50)	44 (19.13)	49 (21.22)	
25.0-29.9	81 (32.70)	61 (27.89)	67 (24.74)	60 (24.90)	
≥ 30	118 (45.15)	138 (52.28)	129 (54.81)	131 (52.11)	
Missin	2 (0.88)	2 (1.33)	5 (1.32)	6 (1.76)	
Diabetes					0.441
No	164 (72.48)	149 (68.54)	163 (73.04)	155 (67.52)	
Yes	82 (27.52)	96 (31.46)	82 (26.96)	91 (32.48)	
CKD					0.028
No	188 (83.14)	182 (74.32)	177 (72.82)	158 (69.63)	
Yes	55 (16.11)	60 (23.95)	64 (26.45)	83 (28.67)	
Missing	3 (0.75)	3 (1.73)	4 (0.73)	5 (1.69)	
Hypertension					0.001
No	75 (41.63)	79 (39.98)	82 (36.29)	68 (26.21)	
Yes	171 (58.37)	166 (60.02)	163 (63.71)	178 (73.79)	
COPD					0.244
No	223 (90.41)	225 (87.44)	219 (88.83)	211 (84.62)	
Yes	23 (9.59)	20 (12.56)	26 (11.17)	35 (15.38)	
CHD					0.095
No	220 (90.03)	224 (91.05)	227 (90.24)	225 (91.44)	
Yes	26 (9.97)	20 (6.76)	17 (9.56)	20 (7.75)	
Missing	0	1 (2.19)	1 (0.19)	1 (0.81)	
Hyperlipidemia					0.068
No	40 (14.13)	43 (14.56)	48 (19.11)	51 (21.79)	
Yes	206 (85.87)	202 (85.44)	197 (80.89)	195 (78.21)	
Klotho (pg/ml)	909.36±390.53	828.83±262.00	818.91±272.46	781.16±256.24	<0.001
Neutrophils count (10^3^/μL)	3.04±0.90	3.87±1.09	4.81±1.31	6.25±2.24	<0.001
Lymphocyte count (10^3^/μL)	2.54±2.47	2.19±0.69	2.00±0.60	1.69±0.68	<0.001
Platelet count (10^3^/μL)	200.48±52.70	233.91±51.49	247.58±50.92	293.54±91.62	<0.001

Abbreviations: BMI: body mass index; CHD: coronary heart disease; CKD: chronic kidney disease; COPD: chronic obstructive pulmonary disease; N: Number of observed; NHANES: National Health and Nutrition Examination Survey; SII: systemic immune-inflammation index. For continuous variables: Mean +/- SD; *P*-value was calculated by weighted linear regression model. For categorical variables: Unweighted N and weighted %; *P*-value was calculated by weighted chi-square test.

**Table 2:** Association of SII with Klotho (pg/ml) in RA patients.

Exposure	Model 1	*P*-value	Model 2	*P*-value	Model 3	*P*-value
	β (95% CI)		β (95% CI)		β (95% CI)	
SII	-6.53 (-10.31, -2.74)	0.0007	-6.14 (-9.93, -2.35)	0.0015	-6.33 (-10.15, -2.53)	0.0011
SII quartiles						
Quartile 1	Reference		Reference		Reference	
Quartile 2	-80.53 (-133.34, -27.73)	0.0029	-76.88 (-129.69, -24.06)	0.0044	-68.27 (-121.14, -15.41)	0.011
Quartile 3	-90.45 (-144.88, -36.02)	0.0011	-86.52 (-140.96, -32.08)	0.0018	-85.60 (-140.08, -31.13)	0.0021
Quartile 4	-128.2 (-181.12, -75.27)	<0.001	-123.22 (-176.46, -69.98)	<0.001	-120.93 (-174.84, -67.02)	<0.001
*P* for trend	<0.001		<0.001		<0.001	

Model 1: no covariates were adjusted. Model 2: age, sex, and race/ethnicity were adjusted. Model 3: age, sex, and race/ethnicity, BMI, diabetes, CKD, hypertension, COPD, CHD, and hyperlipidemia were adjusted. Abbreviations: BMI: body mass index; CHD: coronary heart disease; CI: confidence interval; CKD: chronic kidney disease; COPD: chronic obstructive pulmonary disease; RA: rheumatoid arthritis; SII: systemic immune-inflammation index.

**Table 3:** Subgroup analysis for the association between SII and Klotho (pg/ml) in RA patients.

Parameters	N	β (95% CI)	* P*	*P* for Interaction
Age, years				0.251
40-59	423	-9.3 (-14.5, -4.0)	0.001	
60-69	345	-3.9 (-11.0, 3.3)	0.287	
70-79	214	-1.7 (-10.7, 7.2)	0.703	
Sex				0.585
Male	400	-4.8 (-10.2, 0.7)	0.086	
Female	582	-6.9 (-12.3, -1.5)	0.013	
Race/ethnicity				0.732
Non-Hispanic White	293	-7.4 (-18.6, 3.8)	0.196	
Non-Hispanic Black	366	-14.74 (-12.5, -3.1)	0.001	
Non-Hispanic Asian	162	1.6 (-14.8, 18.0)	0.85	
Others	161	-5.9 (-16.0, 4.2)	0.252	
BMI				0.461
<25	182	-8.7 (-15.0, -2.4)	0.007	
25.0-29.9	269	-5.4 (-11.6, 0.9)	0.094	
≥ 30	516	-4.4 (-12.0, 3.2)	0.26	
Diabetes				0.289
No	631	-5.1 (-9.6, -0.6)	0.026	
Yes	351	-9.6 (-16.7, -2.4)	0.009	
CKD				0.876
No	705	-6.1 (-10.4, -1.9)	0.005	
Yes	262	-8.3 (-17.0, 0.3)	0.058	
Hypertension				0.435
No	304	-9.7 (-18.6, -0.8)	0.034	
Yes	678	-5.8 (-10.1, -1.6)	0.007	
COPD				0.447
No	878	-6.8 (-10.9, -2.8)	0.001	
Yes	104	-2.3 (-13.6, 9.0)	0.692	
Coronary heart disease				0.257
No	896	-6.2 (-10.0, -2.3)	0.002	
Yes	83	-18.6 (-40.3, 3.0)	0.092	
Hyperlipidemia				0.051
No	182	-14.1 (-20.9, -7.3)	0.001	
Yes	800	-3.7 (-8.2, -0.9)	0.112	

Age, sex, and race/ethnicity, BMI, diabetes, CKD, hypertension, COPD, CHD, and hyperlipidemia were all adjusted except the variable itself. Abbreviations: BMI: body mass index; CHD: coronary heart disease; CI: confidence interval; CKD: chronic kidney disease; COPD: chronic obstructive pulmonary disease; N: Number of observed; RA: rheumatoid arthritis; SII: systemic immune-inflammation index.
