# Supplementary material for: Interplay of Systemic Immune-Inflammation Index and Serum Klotho Levels: Unveiling a New Dimension in Rheumatoid Arthritis Pathology
Source: Int J Med Sci. 2024 Jan 1;21(2):396–403. doi: 10.7150/ijms.89569 (PMC10758150; doi:10.7150/ijms.89569)
Supplement: Supplementary file 1 — Supplementary table. [file ijmsv21p0396s1.pdf]

Supplementary Material 1 Characteristics of the SII and Klotho levels in RA and non-RA populations.

RA	Non-RA (N = 12773)	RA (N = 982)	*P*-value
SII	530.1 ± 403.5	600.9 ± 485.2	<0.001
Klotho (pg/ml)	855.7 ± 309.5	840.7 ± 313.6	0.033
Neutrophils count (10^3/μL)	4.2 ± 1.8	4.4 ± 1.9	0.005
Lymphocyte count (10^3/μL)	2.1 ± 0.8	2.1 ± 1.6	0.040
Platelet count (10^3/μL)	242.0 ± 64.5	247.2 ± 72.6	0.141
SII quartiles			<0.001
Quartile 1	3216 (25.2%)	223 (22.7%)	
Quartile 2	3220 (25.2%)	219 (22.3%)	
Quartile 3	3204 (25.1%)	234 (23.8%)	
Quartile 4	3133 (24.5%)	306 (31.2%)	

Abbreviations: BMI: body mass index; CHD: coronary heart disease; CI: confidence interval; CKD: chronic kidney disease; COPD: chronic obstructive pulmonary disease; RA: rheumatoid arthritis; SII: systemic immune-inflammation index.
